# Gestational High-Fat Diet Drives Premature Differentiation of Orexigenic Neurons and Reactivity of Astrocytes in the Fetal Rat Lateral Hypothalamus

**DOI:** 10.3390/brainsci16010052

**Published:** 2025-12-30

**Authors:** Nuria Galindo-Solano, Ximena Trejo-Villarreal, Geovanna Díaz-Olivares, Gustavo Rea-Palomino, Dayna Montes-Aguirre, Maricela Villagrán-Santa-Cruz, Gabriel Gutiérrez-Ospina

**Affiliations:** 1 Laboratorio de Biología de Sistemas, Departamento de Biología Celular y Fisiología, Instituto de Investigaciones Biomédicas, Universidad Nacional Autónoma de México, Ciudad de México 04510, Mexico; nuria.gs@ciencias.unam.mx (N.G.-S.);; 2 Programa de Doctorado en Ciencias Biomédicas, Unidad de Posgrado, Universidad Nacional Autónoma de México, Ciudad de México 04510, Mexico; 3 Laboratorio de Biología Tisular y Reproductora, Facultad de Ciencias, Universidad Nacional Autónoma de México, Ciudad de México 04510, Mexico; 4 Coordinación de Psicobiología y Neurociencias, Facultad de Psicología, Universidad Nacional Autónoma de México, Ciudad de México 04510, Mexico

**Keywords:** developmental origins of health and disease (DOHaD), predictive adaptive phenotype, maternal high-fat diet, fetal programming, hypothalamus, orexins, astrocytes

## Abstract

**Background/Objectives**: Gestational exposure to a high-fat diet (HFD) reprograms hypothalamic orexigenic circuits prenatally. However, whether astrocytes, critical modulators of this system, are also imprinted by HFD in the fetal brain remains unknown. We investigated the impact of HFD on the prenatal neuroglial architecture of the lateral hypothalamic area (LHA). **Methods**: Female Wistar rats were fed a control or a 60% fat diet for 12 weeks. Upon reaching obesity (Lee index ≥ 310), dams were mated. Fetuses were harvested via cesarean section at term, and their brains were processed for immunohistochemistry and morphometry to assess cell proliferation, orexin neuron density, and astrocytic reactivity in the LHA. **Results**: HFD significantly increased cell proliferation and orexinergic neuron density, and induced early signs of astrocyte reactivity in the fetal LHA. These findings reveal that both neuronal and glial components of the LHA orexigenic axis are structurally reprogrammed before birth. **Conclusions**: This study provides the first evidence that HFD simultaneously alters neuronal and glial developmental trajectories in the fetal hypothalamus. The concurrent programming of astrocytes and orexigenic neurons suggests a prenatal origin for neuroinflammatory susceptibility, reframing obesity as a neurodevelopmental disorder shaped by early life nutritional environments.

## 1. Introduction

Eating disorders and their metabolic consequences have traditionally been researched under the assumption that they are acquired during postnatal life. This approach posits a dysregulation of the neural mechanisms controlling food intake, energy expenditure, and body fat deposition, often associated with psycho-affective disorders, inadequate dietary choices, unhealthy cultural eating habits, and/or a sedentary lifestyle. However, emerging evidence suggests that inadequate nutritional exposure during critical gestational periods conditions long-term metabolic programming, predisposing individuals to dysregulated eating behaviors and metabolic diseases [1,2]. A paradigmatic example of this phenomenon is obesity, particularly in its early onset form [3]. The offspring of obese mothers exhibit a significantly higher risk of developing obesity [4], a phenomenon not exclusive to humans. Gestational exposure to hypercaloric diets rich in simple carbohydrates and/or saturated fats also induces persistent alterations in the mechanisms regulating energy homeostasis in rodents and non-human primates [5].

Although epidemiological and experimental studies have clearly established the correlation between increased gestational caloric intake and the offspring’s proclivity for obesity, the underlying mechanisms remain much less clear. In this regard, the Predictive Adaptive Response hypothesis offers a suitable theoretical framework. This hypothesis refers to a form of developmental plasticity wherein cues received in early life influence the development of a phenotype that is normally adapted to the environmental conditions of later life. When the predicted and actual environments differ, the mismatch can have adverse consequences for Darwinian fitness and, subsequently, for health [6]. Recently, Rajamoorthi and colleagues proposed this may indeed be the case for obesity, suggesting that a fetus exposed to hypercaloric diets anticipates a postnatal environment of caloric abundance. If these expectations are met or exceeded after birth, the likelihood of developing obesity and its associated comorbidities increases substantially [7].

But in practical terms, how does the fetus develop a predictive adaptive phenotype? Part of the answer lies in the way brain circuits regulating feeding behavior adapt their anatomical and functional organization during gestation [5]. It is now accepted that the functional dysregulation of key hypothalamic nuclei—particularly the arcuate, lateral, and paraventricular nuclei—can be programmed during intrauterine life [1,2]. Studies in animal models have demonstrated that maternal overnutrition leads to epigenetic modifications in genes that regulate orexigenic neuropeptides such as neuropeptide Y (NPY) and galanin [5]. Notably, Chang and colleagues reported that the offspring of rats exposed to aHFD exhibit hyperphagia and early onset obesity, accompanied by an increased expression of orexigenic neuropeptides. Interestingly, these postnatal changes were preceded by an increased proliferation of the fetal hypothalamic neuroepithelium and a higher number of orexin-immunoreactive neurons. The consequences of this predictive adaptive response, documented shortly after birth, persisted well into adult life [8] and involved the hypothalamic orexinergic system, which modulates appetite, wakefulness, and energy expenditure [9,10].

The traditional approach to unraveling the mechanisms of obesity has focused almost exclusively on neuronal function. However, neuronal activity and metabolism are intrinsically modulated by surrounding astrocytes. These glial cells are crucial for brain homeostasis, regulating neurovascular coupling, synaptic plasticity, neuronal energy metabolism, and the immune response [11]. Accumulated evidence from postnatal studies demonstrates that astrocytes are highly sensitive to metabolic imbalances. For instance, HFD exposure in young and adult mice induces profound morphological and metabolic remodeling in hypothalamic astrocytes, including lipid accumulation and the activation of proinflammatory pathways like the NAD+–CD38 salvage pathway, which directly contributes to obesity development [12,13]. In diet-induced obesity, reactive astrogliosis is observed in the hypothalamus, where astrocytes tonically release GABA, thereby inhibiting neighboring neurons that control energy expenditure [14].

Beyond inflammation, the functional integrity of neuro-astrocytic communication is vital for the circuits that regulate energy homeostasis. Recent investigations have revealed that astrocytes in the lateral hypothalamus are key regulators of orexin neuron firing and physical activity patterns [15]. Indeed, the transport of lactate from astrocytes to orexin neurons is indispensable for maintaining their tonic firing and stabilizing wakefulness [16]. This intimate relationship is reinforced by findings showing that orexin-A can exert neuroprotective effects by attenuating astrocyte activation and apoptosis in neuroinflammatory contexts [17], a condition commonly associated with HFD consumption.

While postnatal HFD alters astrocyte function and a critical functional dyad exists between astrocytes and orexinergic neurons, it remains unknown whether astrocytes are also a target of diet-induced fetal programming. Building on the work of Chang et al. [8], which demonstrated prenatal alterations in orexinergic neuronal development, the question arises as to whether the neuron–astrocyte functional unit is already affected before birth. Therefore, the present study aims to determine whether gestational exposure to an HFD alters the development of hypothalamic circuits that regulate appetite, focusing specifically on the interaction between orexinergic neurons and astrocytes in the LHA. We hypothesize that prenatal exposure to an HFD induces early alterations, already present before birth, in the organization and maturation of both the neuronal and astrocytic populations involved in hunger regulation.

## 2. Materials and Methods

To facilitate understanding of the experimental design, a schematic timeline summarizing all experimental steps (maternal diet exposure, mating, gestational monitoring, fetal collection, placental processing, and hypothalamic analyses) has been included as Supplementary Figure S1.

### 2.1. Animals and Experimental Design

Female Wistar rats (21 days old) born and raised in the Unidad de Modelos Biológicos at the Instituto de Investigaciones Biomédicas, Universidad Nacional Autónoma de México (UNAM), were housed in standard polycarbonate cages with wood-chip bedding and placed in a local colony room, under controlled ambient conditions (21 ± 0.5 °C; 12:12 h light/dark cycle; lights on at 07:00 h). As environmental enrichment, sterile cardboard rolls were provided in each cage and remained available throughout the entire experimental period. All procedures were performed in accordance with the Mexican Official Guideline for the Use and Care of Laboratory Animals (NOM-062-ZOO-1999) and were approved by the Committee for the Care and Use of Laboratory Animals at the Instituto de Investigaciones Biomédicas, UNAM (protocol number: 238)

After a 3-day acclimation period with ad libitum access to food (Teklad Global 18% Protein Rodent Diet; Teklab, Inc., Collinsville, IL, USA) and water, animals were randomly assigned into two groups: control diet (CD; n = 13) and HFD (n = 24). Sample size for CD and HFD groups was estimated based upon a pilot study aimed at evaluating the effects of HFD on pregnancy efficiency (Supplementary Material: Pilot study). Baseline body weight, body length, and fasting glucose concentration (4 h fasting; Accu-Chek Instant, Roche, Indianapolis, IN, USA) were recorded before diet administration. Maternal blood samples for this and all subsequent metabolic tests were withdrawn through tail puncturing. Animals in the HFD group were fed with a 60% fat diet (Envigo TD.06414; Indianapolis, IN, USA) for 12 weeks, while CD animals remained on the standard diet.

Body weight was recorded weekly in both CD and HFD groups throughout the entire experimental process. At the 12th week of treatment, the Lee index was calculated as [cube root of body weight (g)/naso–anal length (cm)] × 1000; a value greater than 310 is considered indicative of obesity in rats [18]. While all CD females were selected for mating and subsequent experimental procedures, only HFD females exceeding this threshold continued in the study. To monitor the metabolic status of CD and HFD, fasting blood cholesterol and triglyceride levels were measured using reactive strips (Roche, Indianapolis, IN, USA). A glucose tolerance test (GTT) was also conducted in animals of both groups that fasted for 8 h. After establishing the blood baseline glucose concentration, an intraperitoneal injection of glucose (1.5 mg/g; Merck Millipore, 1.08337.1000; Burlington, MA, USA) diluted in 0.9% saline (1.5 mL/kg) was administered. Glucose levels were then recorded at 30-, 60-, 90-, and 120-min post-injection [19].

### 2.2. Gestation, Prenatal BrdU Administration, and Placenta and Brain Collection

After selection, all CD (n = 13) and the 22 HFD females that had Lee index > 310 were paired with experienced fertile Wistar males. Pairing was conducted randomly, following the criteria of sexual receptivity phases [20]. Receptive females were housed overnight with a male (2:1 ratio). Pregnancy was confirmed by monitoring body weight gain over 15 days [21]. Non-pregnant CD and HFD females were excluded from the study after three failed mating attempts. This mating protocol resulted in 6 CD and 7 HFD pregnancies, all of which were included in the subsequent analyses.

Following the dietary conditioning period, only HFD females that reached the obesity threshold (Lee’s index ≥ 310) were selected for mating. Females from both CD and HFD groups that failed to conceive after three consecutive mating attempts were excluded from the study. As a result, the final experimental groups included only obese HFD females and successfully pregnant dams, ensuring homogeneity of metabolic status at gestational onset.

To label proliferating cells, all pregnant rats received intraperitoneal injections of 5-bromo-2′-deoxyuridine (BrdU; 10-160, Sigma-Aldrich, St. Louis, MO, USA) at a dose of 20 mg/kg diluted in 7 mM NaOH and 0.9% NaCl every 8 h on gestational day 12, the day when hypocretin ⁄orexin-containing neurons are produced in the developing ventral diencephalon. Body weight and length continued to be monitored throughout pregnancy (days 0–20). On gestational day 20, animals fasted for 8 h, after which cholesterol and triglyceride concentrations were determined and a GTT was conducted [22].

At gestational day 20, all CD and HFD dams were euthanized by using an intraperitoneal overdose of pentobarbital [23]. An abdominal dissection was performed to remove the placentas, which were then weighed. Fetuses (CD: 38 males and 27 females; HFD: 43 males and 39 females) were extracted, sexed, weighed, and their waist circumference and naso–anal length measured. Fetal blood samples were collected and fetal brains dissected after decapitation. Blood glucose concentration was immediately measured using a handheld glucometer (Accu-Chek Instant, Roche, Indianapolis, IN, USA). The remaining blood was centrifuged at 3000 rpm for 10 min at 4 °C, and serum aliquots were stored at −80 °C. Further biochemical analyses of the fetal serum (e.g., cholesterol and triglycerides) were not included in the present study.

### 2.3. Placental Processing and Histology

After excision, CD (n = 6) and HFD (n = 6) placentas were washed with PBS (0.1 M, pH 7.5) and run through two processing protocols. One group underwent cryoprotection through a sucrose gradient (10%, 20%, and 30%, 24 h each) prior to cryosectioning, while the other group followed a standard paraffin-embedding protocol. For paraffin embedding, tissues were dehydrated using a graded alcohol series (30–100%), cleared with alcohol–xylene (1:1) and xylene, followed by xylene–paraffin (1:1), with each step lasting 30 min.

Cryoprotected placentas were sectioned at 20 μm thickness using a cryostat, with five-section intervals and three sections per slide, totaling nine slides per sample. Oil Red O staining (1.02419; Sigma-Aldrich, St. Louis, MO, USA) was performed on these cryosections. Slides were sequentially washed in PBS, incubated in 100% propylene glycol, stained with Oil Red O solution, differentiated in 85% propylene glycol, rinsed in distilled water, and mounted with aqueous medium. This staining allowed for the identification and quantification of lipid droplets. Image acquisition was performed 24 h after staining.

Paraffin-embedded tissues were sectioned at 7 μm thickness using a microtome, arranged in rows of nine slides with 10-section intervals, ensuring three sections per slide. These sections were stained with hematoxylin and eosin (H&E) to analyze tissue histological organization and cell density, while Masson’s trichrome staining was used to evaluate tissue architecture. Quantitative histological analyses were performed exclusively in the labyrinth zone, which is responsible for maternal–fetal exchange. The junctional/spongiotrophoblast and decidual zones were not included [24,25].

### 2.4. Fetal Brain Tissue Processing

After dissection, CD (n = 18) and HFD (n = 18) exposed brains were fixed by immersion in buffered paraformaldehyde (4%) at 4 °C for 24 h and then washed with phosphate-buffered saline (PBS, 0.1 M, pH 7.4) and sequentially cryoprotected in 10%, 20%, and 30% buffered sucrose until they sank in each solution. Lastly, the brains were immersed in a 30% sucrose solution supplemented with 0.01% sodium azide and stored at 4 °C, until use.

For immunohistochemistry, six brains of both groups were used for each antigen. Each brain was embedded in OCT (Tissue-Tek, SAKU4583, Torrance, CA, USA) and cryo-sectioned coronally (50 μm) at −35 °C using a cryostat. Sections were mounted on poly-L-lysine-coated slides (50-279-88, Fisher Scientific, Waltham, MA, USA). The sections were washed in PBS, treated with 0.3% H_2_O_2_ for 10 min, and incubated in permeabilization/blocking buffer (PBS 0.1 M, 1% BSA, and 0.3% Triton X-100) for 30 min at room temperature. BrdU (mouse, mab3510, CHEMICON, Temecula, CA, USA), Glial Fibrillary Acidic Protein (GFAP, chicken, AB4674; ABCAM Waltham, MA, USA), and Orexins A/B (rabbit, AB6214 and AB229714, ABCAM) primary antibodies (1:200) were incubated diluted in blocking buffer for 48 h at 4 °C in a humid chamber. Following three washes, sections were incubated with anti-mouse (1:500, AP192B, Merck-Millipore, Burlington, MA, USA), anti-chicken (1:500, AP194B, Merck-Millipore, Burlington, MA, USA), or anti-rabbit (1:500, AP182B; Merck-Millipore, Burlington, MA, USA) secondary antibodies raised in donkey, diluted in blocking buffer for 2 h at room temperature. After further washes, antigen detection and signal amplification were achieved by consecutively using the ABC kit (PK-6100, VECTOR, Newark, CA, USA) for 90 min, and the 3,3’-diaminobenzidine (DAB) detection kit (SK-4100, VECTOR, Newark, CA, USA) for 30 s based on the protocols recommended by the supplier. Slides were then washed, dehydrated, and mounted with Cytoseal 60 (8310-4, Thermo Scientific, Waltham, MA, USA).

For total cell number estimation, a subset of consecutive sections to those used for immunocytochemistry was used. These sections were stained with 4’,6-diamidino-2-phenylindole (DAPI, 0.1%, CAS 28718-90-3, Merck-Millipore, Burlington, MA, USA), washed in PBS, and coverslipped with Antifade medium (ENZ-53002-M010, Enzo: Plymouth Meeting, PA, USA).

Negative controls were performed in parallel by processing consecutive sections under identical conditions but omitting the primary antibody. These controls showed no staining, confirming the specificity of the immunohistochemical signal. All primary antibodies employed (BrdU, GFAP, and Orexin A/B) have been validated for use in rat brain tissue by manufacturers and previous publications.

Lastly, although fetuses were sexed at collection, sex-separated analyses were performed only for total cell density using DAPI staining. These analyses revealed no statistically significant differences between male and female fetuses at gestational day 20 (see Supplementary Material). Based on these results, male and female fetal brains were pooled for all subsequent immunohistochemical analyses.

### 2.5. Image Acquisition and Analysis

The LHA was anatomically identified and used as the sole region of interest for quantitative analyses. Its anatomical location was established based on the Atlas of the Neonatal Rat Brain [26], and its boundaries defined using the hypothalamic arcuate, dorsomedial, ventromedial, and paraventricular nuclei as neighboring anatomical landmarks. (Supplementary Figure S2). Sections were photographed at 10× and 20× magnification using an Olympus BX51WI microscope equipped with an automated X/Y/Z stage. Brightness, contrast, and gain were kept constant across all samples. Virtual 2D slices were generated using StereoInvestigator software (Version 2025.1.2; MBF Bioscience, Williston, VT, USA). DAPI-stained images were analyzed using ImageJ software (Version 1.54k, National Institutes of Health). Images were scaled, converted to blue channel only, and LHA was manually outlined to calculate area and perimeter. Cells were quantified within these regions using size (0–∞ μm^2^) and circularity (0–1) thresholds. BrdU-, orexin-, and GFAP-labeled cells were quantified using the same procedure. After image scaling and 8-bit conversion, thresholds were set according to cell type: size (3–12 μm^2^) and circularity (0.5–1.0). Quantification of cell number, area, and perimeter was carried out within LHA boundaries. All estimates were conducted by specialized personnel blinded to the experimental condition of the histological material being analyzed. For each technique (DAPI, BrdU, GFAP, and orexin), six random fetuses per experimental group were used to conduct the analysis. All coronal sections containing the LHA were included per fetus. Sections were cut at 50 μm thickness, and an average of 12–15 consecutive slices per fetus were analyzed, spanning the entire rostrocaudal extension of the LHA.

### 2.6. Statistical Analysis

Data distribution was first assessed using the Shapiro-Wilk test. Normally distributed data are presented as the mean ± standard deviation (SD), whereas non-normally distributed data are expressed as the median (interquartile range, IQR). For comparisons between two groups (CD vs. HFD), an unpaired Student’s *t*-test was applied for parametric data, while the Mann-Whitney *U* test was used for non-parametric data. A two-way ANOVA with diet and sex as factors was performed for fetal biometric and metabolic variables, followed by Tukey’s post hoc test when appropriate. Categorical variables were analyzed using the χ^2^ test. A significance threshold of *p* < 0.05 was considered statistically significant. All analyses were performed using GraphPad Prism 9.0 (GraphPad Software, San Diego, CA, USA). Sample sizes (n) for each analysis are indicated in the figure legends and Section 3.

## 3. Results

### 3.1. The Consumption of HFD for 12 Weeks Promotes the Development of an Obese and Glucose-Intolerant Phenotype in Female Rats

HFD consumption led to significant body weight and length increases that progressed throughout the 12-week conditioning period. The differences between CD (n = 13) and HFD (n = 24) females reached statistical significance from weeks 4 (*p* < 0.0001) and 3 onward (*p* = 0.0011), respectively (Supplementary Figure S3a,b). In addition, Lee’s index reached, at the end of the conditioning period, a mean value of 327.5 ± 14.18 and 297.7 ± 5.67 in HFD and CD females (*p* < 0.05), respectively (Supplementary Figure S3c). Notably, 92% of HFD females exhibited Lee index values ≥310, thereby meeting the obesity criterion and qualifying for subsequent phases of the experiment.

The GTT revealed a delayed metabolic response in HFD rats (Supplementary Figure S3d). Indeed, glycemic levels remained significantly higher in HFD females than those in CD ones (*p* < 0.05) at 60, 90, and 120 min post-glucose administration. The area under the curve showed no significant intergroup variation (*p* = 0.7634). Regarding systemic lipid metabolism, no statistically significant differences were found in either serum cholesterol or triglyceride concentrations between HFD and CD females (*p* > 0.05; Supplementary Figure S3e,f).

Of the females initially assigned to the HFD group, only those that fulfilled the obesity criterion and achieved successful pregnancy were included in subsequent analyses. Non-pregnant females, despite reaching the obesity threshold, were excluded after repeated unsuccessful mating attempts. This selection process resulted in a reduced number of pregnancies in the HFD group, which is consistent with the known negative impact of obesity on reproductive efficiency [27].

### 3.2. HFD Exacerbates Metabolic Dysfunction in Already Obese Female Rats’ Ongoing Pregnancy

**Figure 1:**
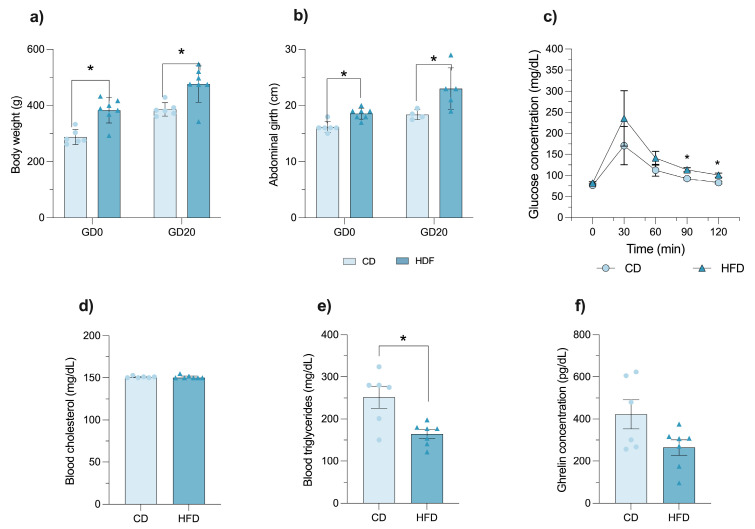
HFD exacerbates metabolic dysfunction in already obese rats during pregnancy. Bar graphs showing (**a**) body weight or (**b**) abdominal girth at gestational day 0 (GD0) and gestational day 20 (GD20). (**c**) Glucose tolerance curve during gestation in rats fed a control diet (CD) or a high-fat diet (HFD), represented as a line graph with error bars. Bar graphs depicting (**d**) blood cholesterol, (**e**) triglyceride, and (**f**) ghrelin levels in CD and HFD females. Data are presented as mean ± SEM for parametric variables and median [IQR] for non-parametric variables. Statistical tests: Two-way ANOVA for (**a**–**c**); Mann–Whitney U test for (**d**), *p* = 0.7634; and Student’s *t*-test for (**e**), *p* = 0.0066, and (**f**), *p* = 0.0586. Asterisks indicate statistically significant differences (* *p* ≤ 0.05). CD, n = 6; HFD, n = 7.

HFD-fed females (n = 7; Lee index ≥ 310) exhibited significantly greater body weight (*p* < 0.0001; Figure 1a) and abdominal girth (*p* < 0.0011; Figure 1b) compared to CD counterparts (n = 6). Post hoc tests confirmed significant differences at gestational day 0 and gestational day 20 (*p* < 0.05).

These changes were accompanied by impaired glucose tolerance in HFD females, who showed significantly higher glycemic levels compared to CD females (*p* < 0.05; Figure 1c). Blood cholesterol levels were similar between CD and HFD females (Figure 1d). However, triglyceride levels were significantly reduced in HFD females (*p* = 0.0066; Figure 1e). Lastly, a non-significant trend toward reduced circulating ghrelin levels was observed in HFD females compared to their CD peers (*p* = 0.0586; Figure 1f).

### 3.3. Maternal HFD Does Not Alter Litter Size or Implantation Sites

**Figure 2:**
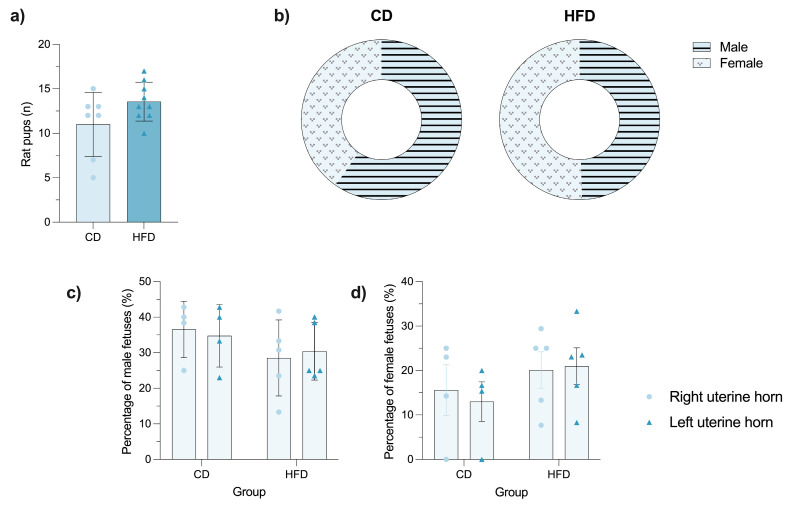
HFD does not impair reproductive efficacy, as litter size, sex ratio, and fetal distribution and implantation preference along the uterine horns remained comparable between control diet (CD) and high-fat diet (HFD) females. (**a**) Bar graph showing the total number of rat pups delivered per litter in CD and HFD females. (**b**) Donut charts representing the sex distribution of offspring delivered by CD and HFD females. Bar graphs showing (**c**) the percentage of fetuses occupying right and left uterine horns, and (**d**) the percentage of fetuses implanted per uterine horn. Data are presented as mean ± SEM. Statistical tests: Student’s *t*-test for (**a**), *p* = 0.1002; two-way ANOVA for (**c**), *p* = 0.1692, and (**d**), *p* = 0.1937. No statistically significant differences were found for any of the parameters. In (**a**,**b**), CD n = 77 and HFD n = 105; in (**c**,**d**), CD n = 8 and HFD n = 10. In panel (**a**), bars represent the mean litter size per group, and circles and triangles indicate individual values. Light-colored bars correspond to the CD, and dark-colored bars correspond to the HFD. In panel (**b**), striped and dotted patterns represent male and female offspring, respectively. In panels (**c**,**d**), circles and triangles denote right and left uterine horns.

Analysis of reproductive parameters revealed no significant differences in the number of offspring per litter between CD and HFD groups (*p* = 0.1002; Figure 2a). Sex distribution analysis (Figure 2b) showed a slight female predominance (60%) in litters from CD dams, while the HFD group exhibited a balanced sex ratio (50:50).

Fetal distribution along the uterine horns showed no significant lateralization bias in either group (*p* = 0.1692), with comparable percentages of fetuses located in the right and left horns (Figure 2c). Similarly, implantation patterns along the longitudinal axis of each horn (anterior, middle, and posterior zones) remained consistent across groups (*p* = 0.1937).

### 3.4. Maternal HFD Induces Placental Lipotoxicity and Structural Alterations

**Figure 3:**
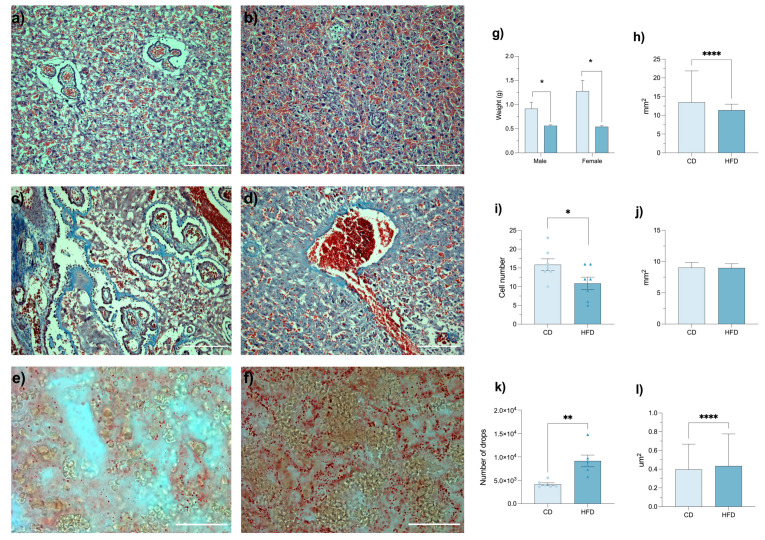
Placental cytoarchitecture deteriorates with the consumption of a high-fat diet (HFD). Photomicrographs showing representative sections of CD (n = 6) and HFD (n = 6) placentas stained with hematoxylin and eosin (**a**,**b**), Masson’s trichrome (**c**,**d**), or Oil Red O (**e**,**f**). A cyto- architectonic disorganization accompanied by lipidic infiltration prevails in placentas exposed to an HFD. Scale bar = 50 µm. Bar graphs depicting placental weight (**g**), surface area (**h**), area occupied by collagen (**i**), trophoblastic cell number (**j**), and the number (**k**) and size (**l**) of oil droplets in CD and HFD placentas. Data are presented as mean ± SEM for parametric variables and median [IQR] for non-parametric variables. Statistical tests: two-way ANOVA for (**g**), *p* < 0.0001; Mann-Whitney U test for (**h**), *p* < 0.0001, (**i**) *p* = 0.9700, and (**j**), *p* < 0.0001; and Student’s *t*-test for (**k**), *p* = 0.0494, and (**l**), *p* = 0.0033. Asterisks indicate statistically significant differences (* *p* ≤ 0.05; ** *p* ≤ 0.01; **** *p ≤* 0.0001).

The placenta is a dynamic organ that adjusts to maternal nutritional and metabolic status, aiming to safeguard fetal development [6]. We then conducted CD and HFD placental histological assessments (Figure 3a–f). Compared to CD placentas, HFD placentas showed a significant reduction in both placental weight (*p* < 0.0001; Figure 3g) and placental surface area (*p* < 0.0001; Figure 3h). The number of trophoblast cells was also significantly reduced in the HFD group (*p* < 0.0001; Figure 3j). In contrast, Masson’s trichrome staining showed no significant difference in the total collagen-stained area (*p* = 0.9700; Figure 3i).

Most notably, Oil Red O staining (Figure 3e,f) confirmed placental lipotoxicity, revealing a marked increase in both the number (*p* = 0.0494; Figure 3k) and size (*p* = 0.0033; Figure 3l) of lipid droplets in HFD placentas. These findings, comparable to previous observations [28,29,30,31], suggest an ongoing process of placental dystrophy in HFD-exposed females. Lipid accumulation may alter placental signaling and metabolic homeostasis, affecting fetal nutrient availability [32].

### 3.5. Maternal HFD Reduces Fetal Body Weight in Both Sexes

**Figure 4:**
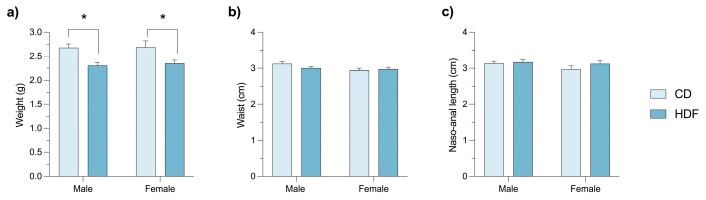
Fetal body weight, regardless of sex, is affected negatively by high-fat diet (HFD) gestational exposure. Bar graphs showing fetal weight (**a**), waist circumference (**b**) and naso-anal length (**c**) by sex in control diet (CD; n = 65) and HFD (n = 82) fetuses. Data are presented as mean ± SEM. Statistical tests: two-way ANOVA for (**a**), *p* < 0.0001, and (**b**), *p* = 0.4631 (**c**). Asterisks indicate statistically significant differences (* *p* ≤ 0.05).

A two-way ANOVA on fetal body weight revealed a significant main effect of maternal diet (*p* < 0.05; Figure 4a), as fetuses from HFD females showed reduced weight compared to CD females. This indicates that gestational HFD impairs tissue accretion rather than overall axial growth, likely by impairing placental nutrient transfer. A significant main effect of sex was also observed (*p* < 0.0001), with male fetuses being consistently heavier than female fetuses across both diet groups. In contrast, no significant effects of diet or sex were found for waist circumference (*p* = 0.4631; Figure 4b) or naso–anal length (*p* = 0.2692; Figure 4c). Comparable reductions in fetal weight, independent of crown-rump length, have been reported [4,33]. These observations suggest that growth sexual dimorphism is maintained under HFD exposure.

Sex-specific analyses were conducted for total cell density in the lateral hypothalamic area using DAPI staining. These analyses revealed no statistically significant differences between male and female fetuses, nor significant diet × sex interactions at gestational day 20 (see Supplementary Material). Based on these findings, data from both sexes were pooled for subsequent analyses.

### 3.6. Maternal HFD Induces Reactive Astrogliosis in the Fetal LHA

**Figure 5:**
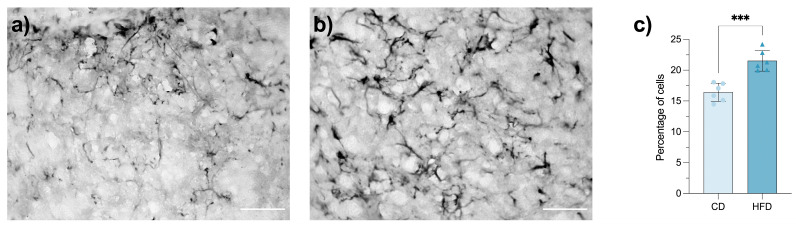
Reactive astrogliosis in the lateral hypothalamic area (LHA) of control diet (CD) and high-fat diet (HFD) fetuses. Representative photomicrographs of glial fibrillary protein (GFAP) immune-stained astrocytes in the LHA of CD (n = 6) (**a**) and HFD (n = 6) (**b**) fetuses. Bar graph (**c**) showing the percentage of GFAP-positive astrocytes in the LHA. Data are presented as mean ± SEM. Statistical test: Student’s *t*-test, *p* = 0.0002. Asterisks indicate statistically significant differences (*** *p* ≤ 0.001). Scale bar = 50 µm. In panel (**c**), bars represent the mean percentage of cells per group, circles indicate individual values from CD samples, and triangles indicate individual values from HFD samples. Light-colored bars correspond to CD and dark-colored bars to HFD.

Maternal obesity leads to increased proliferation and numbers of astrocytes in the developing fetal and neonatal mouse hypothalamus. Here, we revealed that, in CD fetuses, astrocytes display typical resting morphology with fine, filamentous processes and sparse GFAP staining (Figure 5a). In contrast, HFD-exposed fetuses exhibit hypertrophic cell bodies and a dense network of thickened processes. These cellular changes, indicative of reactive astrogliosis, occurred together with a significant increment in the percentage of LHA, GFAP-positive astrocytes (*p* = 0.0002; Figure 5c). The reactive astrocytic phenotype itself suggests an early progression of neuroinflammatory processes, consistent with prior evidence that maternal obesity promotes gliosis in the offspring brain [13].

### 3.7. Maternal HFD Increases Diencephalic Precursor Cell Proliferation and the Percentage of Orexinergic Neuron Density in the Fetal LHA

**Figure 6:**
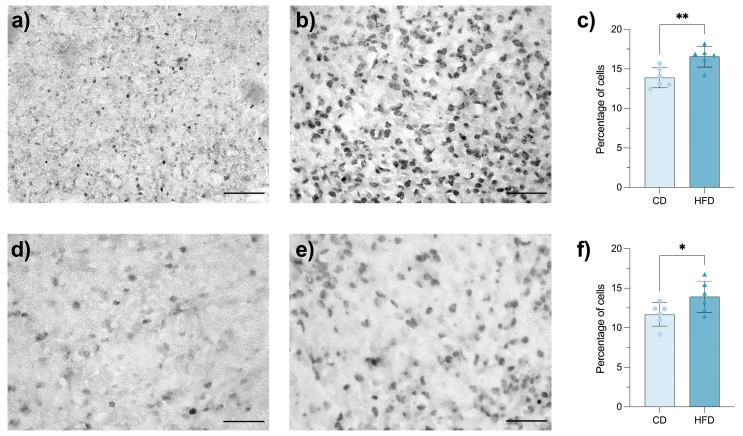
Maternal high-fat diet (HFD) increases diencephalic precursor cell proliferation and the percentage of orexinergic neuron density in the fetal lateral hypothalamic area (LHA). Representative photomicrographs showing cell nuclei labelled as positive for 5-bromo-2-deoxyuridine (BrdU) in the LHA of CD (n = 6) (**a**) and HFD (n = 6) (**b**) fetuses. Bar graph (**c**) showing the percentage of BrdU-positive cell nuclei in the LHA of CD and HFD fetuses. Representative photomicrographs showing neurons labelled as positive for orexin in the LHA of CD (**d**) and HFD (**e**) fetuses. Bar graph (**f**) showing the percentage of orexin-positive neurons in the LHA of CD and HFD fetuses. Data are presented as mean ± SEM. Statistical tests: Student’s *t*-test for (**c**), *p* = 0.0050, and (**f**), *p* = 0.0489. Asterisks indicate statistically significant differences (* *p* ≤ 0.05; ** *p* ≤ 0.01). Scale bar = 50 µm.

Gestational HFD increases the proliferation of hypothalamic neuronal precursors and the postnatal density of orexinergic neurons in the perifornical lateral hypothalamus, a predictive adaptive phenotypic response observed soon after birth and persisting postnatally well into adulthood [8]. Here, we revealed that both cell biological processes take place in the LHA before birth. Indeed, BrdU-positive nuclei increased, qualitatively and quantitatively, significantly in the LHA of HFD fetuses as compared with CD fetuses (Figure 6a–c; *p*= 0.0050). Coincidently, the percentage of orexin immune-stained neurons in LHA was also found to be significantly increased in HFD fetuses (Figure 6d–f; *p* = 0.0489). Heightened proliferation and orexinergic phenotype expression indicate that HFD creates an environment conducive to modified neuronal specification, possibly mediated by increased lipid availability and proinflammatory cytokines crossing the placenta [28].

## 4. Discussion

The present study provides the first evidence that gestational exposure to a high-fat diet (G-HFD) simultaneously programs both neuronal and glial populations in the fetal lateral hypothalamic area (LHA). We demonstrated that, at term, fetuses exposed to HFD exhibit a trio of alterations in the LHA not seen in controls: (1) a significant increase in precursor cell proliferation, (2) a higher density of orexinergic neurons, and (3) clear signs of reactive astrogliosis. These findings confirm our hypothesis that HFD alters the hypothalamic neuroglial architecture before birth, laying a neurobiological foundation for later metabolic dysfunction.

The most novel finding of this study is the identification of precocious reactive astrogliosis. While postnatal hypothalamic astrogliosis is a well-documented feature of diet-induced obesity [12,13], our data demonstrate that this glial response is initiated before birth. This is critically important. Astrocytes are not mere support cells; they are active modulators of neuronal metabolism and synaptic plasticity. In the LHA, they are key regulators of orexin neuron activation [15,16]. The presence of reactive astrocytes in the fetal brain suggests that the LHA is not only being programmed but may already be in a state of low-grade stress or neuroinflammation. This early glial activation could fundamentally alter circuit maturation, affecting the organization of the metabolic network during a critical developmental window.

Our findings on the neuronal side confirm and extend the seminal work of Chang et al. [8]. They were the first to report that gestational HFD increased fetal hypothalamic neuroepithelial proliferation and the number of orexin neurons. Our results validate these findings and localize these changes within defined regions of the LHA. This overproduction of orexinergic neurons aligns perfectly with the Predictive Adaptive Response hypothesis [6,7]: the fetus, anticipating a postnatal environment of caloric abundance, constructs a “reinforced” orexigenic circuit to promote food-seeking, preference for fat, and energy storage (see also [7,8]).

An apparent paradox emerging from our data is that HFD-exposed fetuses exhibit reduced body weight, consistent with fetal growth restriction, while simultaneously displaying increased orexinergic neuron density in the LHA. According to the Developmental Origins of Health and Disease framework, an adverse intrauterine environment characterized by placental dysfunction and impaired nutrient transfer constitutes a powerful programming signal, shaping developmental trajectories in anticipation of postnatal conditions [34]. Under this context, fetal growth restriction does not contradict the concept of predictive adaptive programming. In fact, enhanced orexinergic differentiation may represent a compensatory neurodevelopmental response to intrauterine energetic adversity rather than a direct anticipation of caloric abundance. Orexin neurons are highly sensitive to metabolic status and are activated under conditions of fasting and nutrient deprivation, where they promote arousal, food-seeking behavior, and energy mobilization [35]. Thus, increased orexin signaling in HFD-exposed fetuses may reflect an adaptive response to perceived energetic insufficiency during gestation. This interpretation is consistent with previous reports showing that gestational high-fat diet exposure increases hypothalamic orexigenic neuron development before birth, programming neural circuits involved in feeding behavior and energy balance [8]. When such prenatal adaptations occur in response to intrauterine adversity but are followed by postnatal exposure to caloric abundance, a developmental mismatch may arise, predisposing individuals to hyperphagia and metabolic dysregulation later in life [36,37]. Therefore, fetal growth restriction and enhanced orexinergic programming are not mutually exclusive but instead represent complementary components of an adaptive neurodevelopmental response to maternal metabolic dysfunction.

However, the key insight from our study is that these two events—reactive astrogliosis and orexinergic differentiation—are not isolated but concurrent. Recent literature has highlighted the intimate functional relationship between LHA astrocytes and orexin neurons, where astrocytic lactate is vital for neuronal firing [16], and orexin-A can attenuate astrocyte activation [17]. Our data suggest that G-HFD destabilizes this neuro-glial functional unit from its inception. It remains to be determined whether astrocytic reactivity drives the altered neuronal differentiation, or if both cell populations are responding independently to a common lipotoxic or inflammatory signal.

Although sex is a critical determinant of neurodevelopment, the absence of sex differences in total cell density at GD20 suggests that gestational high-fat diet exposure primarily affects early cellular processes that precede overt sexual dimorphism in hypothalamic metabolic circuits [38,39]. At this developmental stage, these circuits are still undergoing differentiation, and sex-dependent divergence is expected to emerge predominantly postnatally. Thus, pooling sexes in the present prenatal analysis does not compromise the interpretation of diet-induced neuroglial programming.

The pathway for this brain programming was made evident by our maternal and placental data. Indeed, we validated that our HFD dams were not only obese before mating (Section 3.1) but remained metabolically dysfunctional during gestation, with impaired glucose tolerance (Section 3.2) and placental dystrophy and lipotoxicity (Section 3.4). Both findings provide a clear mechanistic bridge for the transfer of maternal metabolic stress to the fetus. Although HFD consumption is classically associated with hypertriglyceridemia, obese pregnancies feature maternal triglyceride levels that are unchanged or even decrease during late gestation. This phenomenon is attributed to gestation-specific lipid redistribution, where triglycerides are preferentially diverted toward placental and fetal hepatic compartments rather than remaining in maternal circulation [40,41,42]. Consistent with this interpretation, enhanced placental lipid uptake and storage occur when pregnant women are overnourished, supporting a model in which reduced circulating triglycerides coexist with placental lipotoxicity [28,29]. Thus, the decrease in maternal triglycerides observed at GD20 in our HFD dams is not contradictory to metabolic dysfunction but rather reflects altered lipid trafficking at the maternal–placental interface.

Interestingly, this adverse intrauterine environment resulted in fetuses with lower body weight (Section 3.5), an effect we isolated from the confound of litter size (Section 3.3). This finding is fundamental: it suggests HFD fetuses are not simply “overfed” but are experiencing a lipotoxic and inflammatory environment that is, in fact, malnourishing and growth-restricting. This combination of growth restriction with an over-programmed appetite circuit (orexin) is a potent recipe for postnatal metabolic dysregulation.

This study has several limitations. Our analyses of astrocytes and neurons were morphological and immunohistochemical; we did not measure functional outcomes such as gliotransmitter release, GABAergic neuronal silencing [14], or neuronal firing. Our focus was exclusively on the LHA; it is likely that other nuclei (such as the arcuate) are also programmed. Finally, although we justified pooling the sexes to maximize the statistical power of these exploratory neurobiological findings, future studies must investigate whether this programming is sex-specific, given the known sexual dimorphism in metabolism.

## 5. Conclusions

In conclusion, this study provides the first evidence that HFD imprints a pathological signature on the fetal LHA, characterized by the simultaneous activation of astrocytes and the precocious differentiation of orexinergic neurons. This shifts the paradigm of obesity from being merely a postnatal inflammatory disorder to being a neurodevelopmental disorder, where disease susceptibility is “wired” into the fetal brain, with astrocytes as a key player in this adverse programming from the very beginning.

## Data Availability

The original contributions presented in this study are included in the article/Supplementary Material. Further inquiries can be directed to the corresponding author.

## Supplementary Materials

The following supporting information can be downloaded at https://www.mdpi.com/article/10.3390/brainsci16010052/s1.
