# Supplementary material for: Gestational High-Fat Diet Drives Premature Differentiation of Orexigenic Neurons and Reactivity of Astrocytes in the Fetal Rat Lateral Hypothalamus
Source: Brain Sci. 2025 Dec 30;16(1):52. doi: 10.3390/brainsci16010052 (PMC12839396; doi:10.3390/brainsci16010052)
Supplement: Supplementary file 1 [file brainsci-16-00052-s001.zip › brainsci-4030650-supplementary figure.pdf]

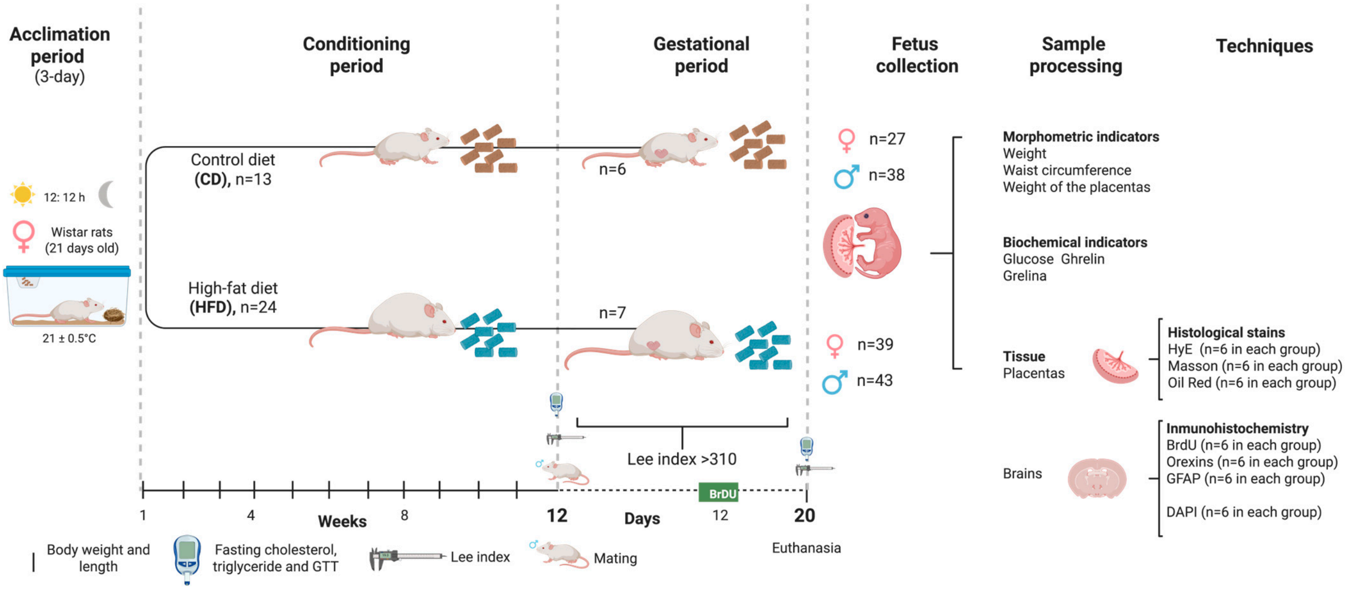

Supplementary Figure S1. Schematic representation of the experimental design. Female Wistar rats (21 days old) were acclimated for 3 days under controlled temperature and light/dark cycles. Animals were randomly assigned to control diet (CD, n = 13) or high-fat diet (HFD, n = 24) groups for a 12-week conditioning period. Body weight and body length were monitored weekly. At week 12, the Lee index was calculated, and females with values ≥ 310 in the HFD group, together with all CD females, were selected for mating. Gestational day 20 fetuses and placentas were collected for somatometric, biochemical, histological, and immunohistochemical analyses. Placental analyses included H&E, Masson's trichrome, and Oil Red O staining, whereas hypothalamic analyses focused on the LHA using BrdU, GFAP, orexin, and DAPI labeling.

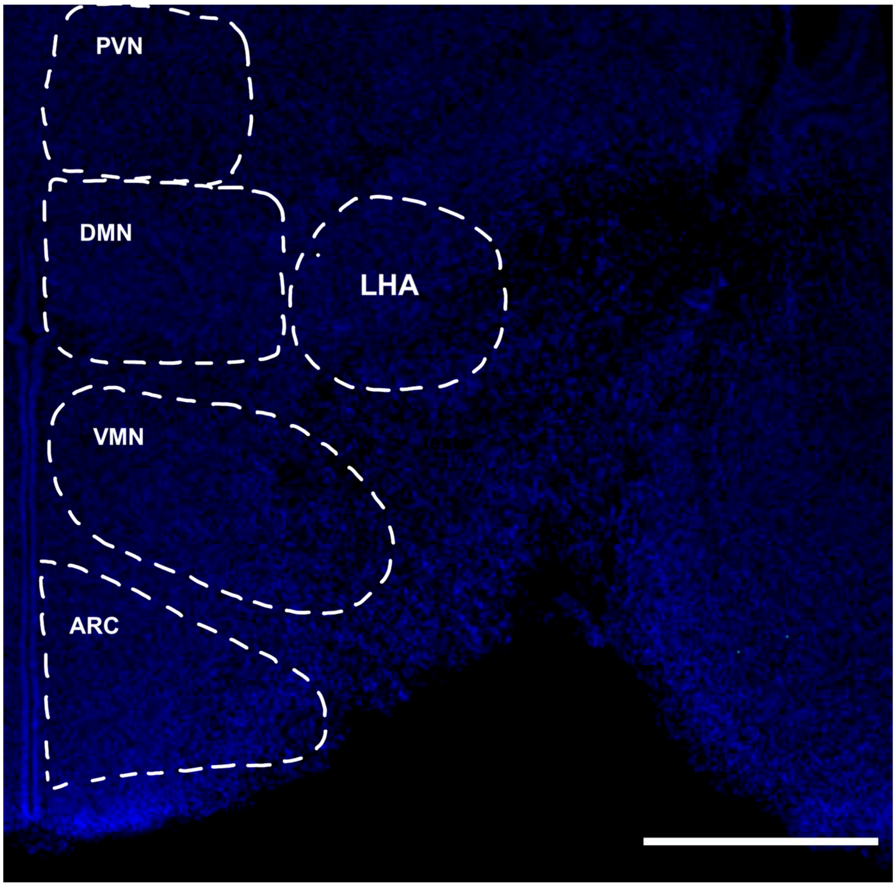

Supplementary Figure S2. Representative fluorescence image showing DAPI nuclear staining in a coronal section of a fetal brain. Dashed lines indicate the anatomical boundaries of the lateral hypothalamic area (LHA), arcuate nucleus (ARC), dorsomedial hypothalamic nucleus (DMH), ventromedial hypothalamic nucleus (VMH), and paraventricular nucleus (PVN). **Note:** ARC, DMH, VMH, and PVN are shown only to provide anatomical reference for the location of the LHA; quantitative analyses were performed exclusively in the LHA. Scale bar = 500 μm.

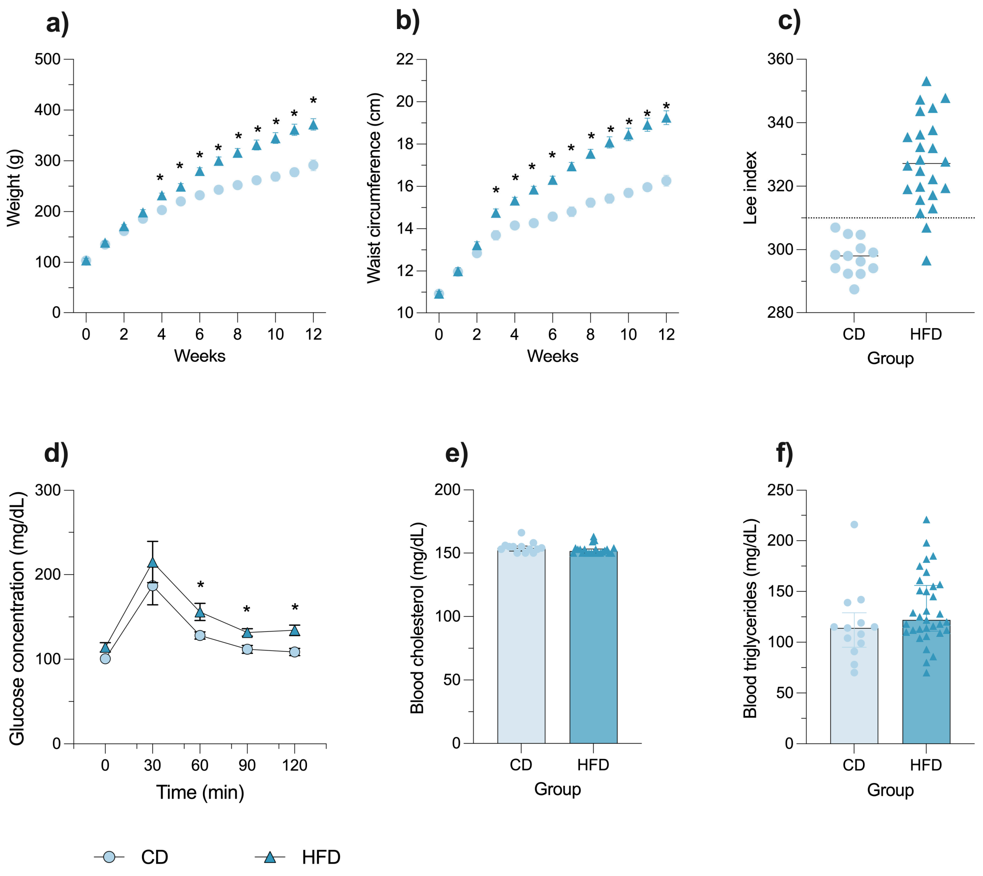

Supplementary Figure S3. Physiological and metabolic parameters in female rats following HFD exposure. (**a**) Body weight showing a significant increase in the HFD group from week 6 of treatment. (**b**) Body length showing a significant increase from week 3. (**c**) Lee index at 12 weeks, confirming the establishment of obesity in HFD females (≥310). (**d**) Glucose tolerance curve showing delayed glucose clearance in the HFD group at 60, 90, and 120 min post-administration. (**e**) Blood cholesterol levels. (**f**) Blood triglyceride levels, with no significant differences between groups. Data are presented as mean ± SEM for parametric variables and median [IQR] for non-parametric variables. Statistical tests: two-way ANOVA for (**a**) $p < 0.0001$, (**b**) p < 0.0001, and (**d**) $p = 0.0148$. Student's *t*-test for (**c**) $p < 0.0001$. Mann–Whitney *U* test for (**e**) $p = 0.0759$ and (**f**) $p = 0.0843$. Asterisks indicate statistically significant differences. CD, n = 13; HFD, n = 24.
